# The influence of the chemical composition of essential oils of *Clausena lansium* seeds on the growth of *Candida* strains

**DOI:** 10.1038/s41598-021-99188-x

**Published:** 2021-10-04

**Authors:** Yinzheng Ma, Yuanxiao Wang, Xia Zhou, Heng Yang, Huixin Zhang, Wenhan Chen, Haiying Zhang, Yunxia Zhang, Xiaowen He

**Affiliations:** 1 Public Research Laboratory, Hainan Medical University, Haikou, 571199 China; 2 School of Public Health, Hainan Medical University, Haikou, 571199 China; 3 School of Parmacy, Hainan Medical University, Haikou, 571199 China; 4 Hainan Provincial Key Laboratory of Carcinogenesis and Intervention, Hainan Medical University, Haikou, 571199 China; 5 Key Laboratory of Emergency and Trauma of Ministry of Education, Research Unit of Island, Emergency Medicine of Chinese Academy of Medical Sciences, Hainan Medical University, Haikou, 571199 China

**Keywords:** Antimicrobials, Fungi, Drug discovery and development

## Abstract

*Clausena lansium* (Lour.) Skeels seeds have been shown to have diverse beneficial medical value due to their unique active components. This study analysed the composition of essential oils (EOs) of *C. lansium* seeds and investigated their potential antifungal effects against *Candida* strains*.* A total of forty-six components were identified in all samples by gas chromatography-mass spectrometry (GC–MS). The main components were sabinene, *β*-phellandrene and 4-terpineol. Thirteen EOs of *C. lansium* seeds were classified into three clusters based on their components. Cluster analysis showed that the difference between the tropics and subtropics was the greatest. These EOs and the three main chemicals showed different antifungal activities against five *Candida* species (*C. albicans*, *C. tropicalis*, *C. glabrata*, *C. krusei* and *C. parapsilosis*). The antifungal activity against *C. glabrata* and *C. krusei* was higher than that against other *Candida* strains. EOs of *C. lansium* seeds displayed noteworthy antifungal activity against both sensitive and fluconazole-resistant strains, with inhibition zone diameters in the range of 9.4–23.4 mm. Comprehensive analysis illustrated the importance of sabinene, *β*-phellandrene and 4-terpineol to antifungal activity, and there may be some synergistic effects with other components. These results represent the first report about the correlation between the chemical composition of EOs of *C. lansium* seeds and antifungal activity. Taken together, the results obtained provide scientific evidence for the traditional use of *C. lansium* seeds waste.

## Introduction

Invasive fungal infections have been associated with significant morbidity and mortality in clinical infections^1,2^. Recently, candidiasis has become one of the most common fungal infections, and its incidence has increased rapidly^.^^3–5^. Although *Candida albicans* is the main yeast species causing infections, non-*albicans Candida* species, including *Candida glabrate*, *Candida krusei*, *Candida parapsilosis* and *Candida tropical*, have caused an increasing number of infections over the past decade^6,7^. With a flush pipeline of investigational antifungals, the clinician must identify appropriate roles for currently available therapies. However, many antifungal agents have been found to have different degrees of side effects including allergic reactions, hepatotoxicity, myelosuppression, nervous system disorder, etc.^8–10^. Early on the 80’s, fluconazole (FLZ) became one of the most efficient agents to treat *Candida* infections due to its broad-spectrum activity, high bioavailability, strong tissue penetration, significant curative effect and fewer adverse reactions. It was extensively used on oral candidiasis in HIV patients and with the extensive application of FLZ, there has been a notable increase in drug resistance among infectious yeast species^6,7^. Thus, many efforts have been made to isolate pure natural products for medicinal use.

Natural plant components, especially essential oils (EOs), have attracted much attention from the scientific community^11^. EOs are secondary metabolites found in many plants. They may exist in fruits, seeds, flowers and leaves. They have many beneficial biological activities, such as antioxidant and antimicrobial, anti-inflammatory, antitumour, and analgesic activities. They have already become a focus because of their advantages such as their abundance, low toxicity, broad-spectrum activities and diverse mechanisms of action^12–14^.

*Clausena lansium* (Lour.) Skeels is a tropical species of the Rutaceae family and is widely distributed in China, in provinces such as Hainan, Guangxi, and Guangdong^15^. Almost all parts of the plant, including the fruit, root, bark, leaf, peel, seed and pulp, have been reported to have medicinal values^16–18^. Hainan Province, a subtropical and tropical region of China, is one of the main geographic regions for growing *C. lansium*. In Hainan, *C. lansium* fruits have been used for treating digestive disorders, gastrointestinal diseases and bronchitis diseases, along with the pericarps and seeds^19^. A likely mechanism is that EOs from *C. lansium* fruits can help release gastrointestinal gas, eliminate stagnation, dissipate heat and relieve pain. The pulp of *C. lansium* can be eaten fresh or made into preserved fruit or jam, but the weight of seeds in the fruit is high and is the residual product of pulp processing, resulting in not only a waste of resources but also pollution of the environment. It is a current focus make use of waste seeds to achieve their maximum application value.*C. lansium* seeds contain volatile components, alkaloids, and coumarins^20,21^. They have attracted great attention due to their extensive biological activities, including anti-inflammatory, anticancer, insecticidal, antidiabetic, and antioxidant effects^22,23^. Considering the unique active components in *C. lansium* seeds and their medicinal and agricultural value, increasing research on *C. lansium* seeds has been carried out in recent years. In a previous study, EOs of *C. lansium* seeds from different areas were obtained and analysed^24–27^. However, there has been no comparative study on the components and contents of EOs of *C. lansium* seeds from different areas. Moreover, the correlation between EOs of *C. lansium* seeds and antifungal activity has not been reported.

The antifungal activity of EOs is closely related to their components and contents. The aim of the current study was to comparatively analyse the chemical components of EOs of *C. lansium* seeds of different cultivars and from different areas in Hainan and to investigate the in vitro antifungal activity against *Candida* species, including *C. albicans*, *C. glabrata*, *C. krusei*, *C. parapsilosis* and *C. tropicalis*. The correlation between EOs of *C. lansium* seeds and the antifungal activity was studied. The results of this study may provide support for further study of the resource development and utilization of *C. lansium* seeds in Hainan.

## Results

### Extraction results and chemical composition of Eos

**Figure 1:**
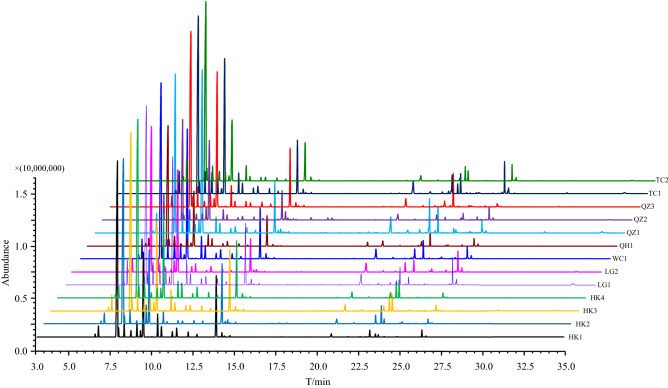
GC–MS chromatograms of EOs of *C. lansium* seeds. (^a^T: retention time).

*C. lansium* EOs extracted by hydrodistillation were white to light yellow in colour. The yields ranged from 0.43 to 0.91% (*v/w*). The colour and yield of EOs are listed in Table 1. There were some differences in the colour and yield of the eleven EOs, which may be related to the geographic source and cultivar, but it is uncertain which is the most important of these factors. Through the statistical analysis, there were no significant differences (*P* > 0.05)*.* It is impossible to come to a conclusion from the colour and yield of EOs. To compare the compositional differences, each EO was subjected to gas chromatography-mass spectrometry (GC–MS) for analysis. Figure 1 shows the GC–MS chromatograms of EOs of *C. lansium* seed. The chemical composition and content results are shown in Table 2.

**Table 1:** Extraction results of EOs of *C. lansium* seeds.

No	Colour	Yield ^a,^ * (%, *v*/*w*)
HK1	White	0.71 ± 0.06
HK2	White	0.78 ± 0.04
HK3	White	0.78 ± 0.01
HK4	White	0.69 ± 0.05
LG1	Light yellow	0.81 ± 0.03
LG2	Light yellow	0.61 ± 0.02
WC1	Light yellow	0.91 ± 0.02
QH1	Light yellow	0.68 ± 0.02
QZ1	White	0.43 ± 0.03
QZ2	Light yellow	0.47 ± 0.04
QZ3	Light yellow	0.84 ± 0.02
TC1	White	0.64 ± 0.01
TC2	Light yellow	0.68 ± 0.06

^a^Values represent the means of three independent replicates ± SD, *P = 0.07.

**Table 2:** Chemical components and contents (%) of EOs of *C. lansium* seeds.

No	Components	Molecular Formula	RI^a^	HK1	HK2	HK3	HK4	LG1	LG2	WC1	QH1	QZ1	QZ2	QZ3	TC1	TC2
1	*α*-thujene	C_10_H_16_		0.39	0.34	0.45	0.43	0.40	0.28	0.44	0.49	0.21	0.33	0.33	0.32	0.59
2	pinene	C_10_H_16_		1.49	1.51	1.98	1.48	2.62	2.00	2.27	1.53	2.04	5.10	1.22	2.30	3.20
3	benzaldehyde	C_7_H_6_O		0.03	0.09	–	0.02	–	0.06	0.05	0.08	0.03	0.28	0.05	–	–
4	sabinene	C_10_H_16_		50.01	51.09	47.37	49.85	39.42	43.20	42.27	52.16	27.70	33.24	42.10	37.92	54.28
5	*β*-pinene	C_10_H_16_		1.05	0.90	1.17	1.19	0.99	0.74	1.04	1.02	0.65	0.69	1.01	0.97	1.49
6	6-methyl-5-hepten-2-one	C_8_H_14_O		–	–	–	–	–	–	0.02	–	–	0.03	–	–	–
7	myrcene	C_10_H_16_		2.15	1.89	2.52	2.56	2.33	1.86	2.21	1.91	2.12	1.94	2.26	2.27	2.01
8	*α*-phellandrene	C_10_H_16_		0.94	0.49	1.32	0.73	2.51	0.92	1.72	0.43	2.14	1.35	0.68	2.36	1.44
9	*α*-terpinene	C_10_H_16_		2.07	0.77	1.69	1.33	1.74	0.37	1.82	1.53	0.94	1.23	1.57	1.46	1.37
10	*p*-cymene	C_10_H_14_		0.94	2.95	1.18	1.33	0.96	1.99	0.51	0.67	1.69	2.85	1.07	0.59	0.76
11	*β*-phellandrene	C_10_H_16_		16.19	14.60	16.12	14.97	21.35	25.16	23.81	15.36	33.24	25.91	25.44	23.94	13.39
12	benzeneacetaldehyde	C_8_H_8_O		0.03	0.03	0.04	0.04	0.04	–	0.06	–	0.03	0.06	0.04	0.04	0.04
13	*β*-*cis*-ocimene	C_10_H_16_		0.06	0.03	0.11	0.11	0.09	0.03	0.07	0.04	0.06	–	0.09	0.10	0.07
14	*γ*-terpinene	C_10_H_16_		3.37	1.81	2.85	2.31	2.82	0.89	2.72	2.47	1.79	2.00	2.69	2.30	2.30
15	3-carene	C_10_H_16_		1.54	0.29	1.03	2.06	1.27	1.32	1.63	1.59	1.08	1.00	0.66	1.15	0.64
16	terpinolene	C_10_H_16_	859	0.80	0.47	0.74	0.61	0.76	0.26	0.66	0.55	0.49	0.45	0.69	0.59	0.53
17	*trans*-4-thujanol	C_10_H_18_O	887	1.44	0.48	0.90	1.60	1.14	1.03	1.25	1.04	0.85	0.90	0.61	0.97	0.66
18	4-terpineol	C_10_H_18_O	977	11.28	11.83	9.92	9.37	8.24	6.33	7.35	7.94	6.66	6.98	8.82	7.07	7.27
19	melilotal	C_9_H_10_O	980	–	0.08	–	–	–	0.06	–	0.05	–	0.19	–	–	–
20	cryptone	C_9_H_14_O	984	0.07	0.35	0.15	0.09	0.15	0.37	0.09	0.04	0.36	1.77	0.11	0.09	–
21	*α*-terpineol	C_10_H_18_O	989	0.67	0.62	0.63	0.61	0.68	0.41	0.63	0.37	0.45	0.44	0.53	0.50	0.60
22	*cis*-piperitol	C_10_H_18_O	1006	0.20	0.25	0.24	0.17	0.18	0.10	–	0.11	0.16	0.15	0.21	0.14	0.13
23	4-isopropylbenzaldehyde	C_10_H_12_O	1038	–	0.05	–	–	–	–	–	–	0.03	0.49	–	–	–
24	( +)-carvone	C_10_H_14_O	1042	–	–	–	–	–	–	–	–	–	0.06	–	–	–
25	phellandral	C_10_H_16_O	1074	–	0.03	–	–	–	–	–	–	0.04	0.41	–	–	–
26	3-methylacetophenone	C_9_H_10_O	1079	–	–	–	–	–	–	–	–	–	0.05	–	–	–
27	2-caren-10-al	C_10_H_14_O	1083	–	0.07	–	–	–	–	–	–	0.05	0.40	–	–	–
28	thymol	C_10_H_14_O	1098	–	0.11	–	–	–	–	–	–	–	0.06	–	–	–
29	*β*-elemene	C_15_H_24_	1194	–	0.04	–	0.04	–	–	–	0.93	0.05	–	0.05	0.09	0.08
30	*cis*-jasmone	C_11_H_16_O	1197	–	–	–	0.05	–	–	–	–	0.09	–	–	–	–
31	caryophyllene	C_15_H_24_	1227	0.74	1.11	1.22	1.12	2.19	2.44	1.80	1.67	2.83	1.86	1.53	2.28	1.26
32	humulene	C_15_H_24_	1162	0.07	0.21	0.19	0.18	0.30	0.29	0.21	0.14	0.50	0.17	0.17	0.35	0.13
33	*γ*-muurolene	C_15_H_24_	1287	0.07	–	–	–	0.27	0.15	0.08	0.05	0.45	0.08	0.20	0.21	0.04
34	*γ*-elemene	C_15_H_24_	1300	0.07	–	0.05	0.04	0.44	–	0.11	0.15	0.08	0.03	0.11	0.26	–
35	*α*-farnesene	C_15_H_24_	1308	0.05	–	0.07	0.07	0.27	–	0.20	0.25	–	–	0.14	0.29	0.41
36	*β*-bisabolene	C_15_H_24_	1311	0.96	1.34	0.68	0.73	1.93	1.52	1.20	0.95	4.29	1.00	0.72	2.05	–
37	*β*-sesquiphellandrene	C_15_H_24_	1328	0.40	2.67	2.18	2.11	0.12	0.04	0.06	0.04	0.26	–	0.18	1.06	2.02
38	*α*-bergamotene	C_15_H_24_	1337	0.29	0.63	2.07	2.35	0.81	1.78	2.20	2.71	2.94	0.46	4.10	2.01	1.45
39	*α*-bisabolene	C_15_H_24_	1347	–	–	–	–	–	–	–	–	0.06	–	0.03	0.03	–
40	*trans*-nerolidol	C_15_H_26_O	1365	–	–	0.03	0.02	0.04	0.03	–	0.03	0.04	0.03	–	0.06	–
41	spathulenol	C_15_H_24_O	1383	–	0.10	0.06	–	0.15	0.19	0.05	0.20	0.47	0.40	0.15	0.13	–
42	caryophyllene oxide	C_15_H_24_O	1390	–	0.25	0.09	0.03	0.05	0.52	0.18	0.22	0.36	1.37	0.12	0.11	–
43	*cis*-*α*-santalol	C_15_H_24_O	1486	0.92	0.56	0.67	0.56	2.60	3.11	1.29	1.39	1.53	2.15	0.36	3.11	1.98
44	*α*-bisabolol	C_15_H_26_O	1489	–	0.22	0.23	0.19	0.21	–	0.08	0.15	–	0.19	0.18	–	0.09
45	*α*-santalol	C_15_H_24_O	1502	0.13	0.10	0.08	0.08	0.52	0.71	0.29	0.36	0.19	0.32	0.04	0.49	0.41
46	*n*-hexadecanoic acid	C_16_H_32_O_2_	1753	–	0.06	0.12	–	–	0.06	–	–	0.15	–	0.05	0.10	–
Total peak number				39	50	46	46	49	50	43	43	66	65	49	53	39
Total identified peak number				30	39	37	37	36	33	35	36	39	39	36	39	31
Total identified peak area percentage				98.42	98.42	98.15	98.43	97.59	98.22	98.37	98.62	97.10	96.42	98.31	97.71	98.64
Monoterpene hydrocarbons				81.00	77.14	78.53	78.96	77.26	79.02	81.17	79.75	74.15	76.09	79.81	76.27	82.07
Oxygen-containing monoterpenes				13.66	13.87	11.84	11.84	10.39	8.30	9.32	9.55	8.60	11.90	10.28	8.77	8.66
Sesquiterpene hydrocarbons				2.65	5.96	6.46	6.60	6.33	6.22	5.86	5.96	11.41	3.60	7.18	8.54	5.31
Oxygen-containing sesquiterpenes				1.05	1.23	1.16	0.88	3.57	4.56	1.89	2.35	2.59	4.46	0.85	3.90	2.48
Other compounds				0.06	0.22	0.16	0.15	0.04	0.12	0.13	1.01	0.35	0.37	0.19	0.23	0.12

^a^RI: calculated retention index; –: Not detected.

**Figure 2:**
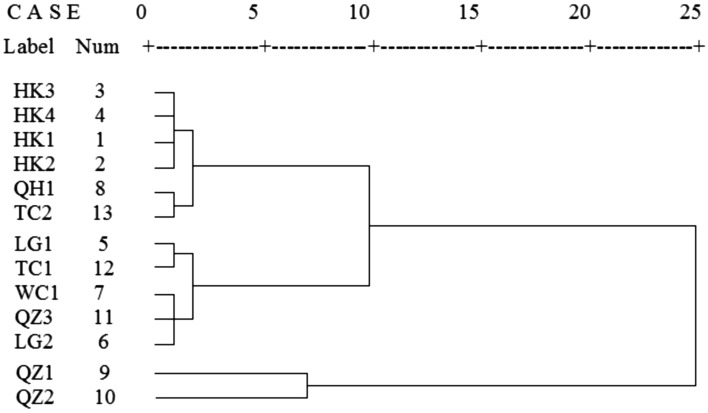
Dendrograms based on the chemical components and contents of EOs of *C. lansium* seeds.

According to the GC–MS analysis, almost thirty components were identified in each EO, amounting to 97.10% to 98.64% of the total components. Differences existed in the components and contents of EOs of *C. lansium* seeds from different cultivars and different areas in Hainan*.* These components were monoterpene hydrocarbons, oxygen-containing monoterpenes, sesquiterpene hydrocarbons and other compounds. The main components were monoterpene hydrocarbons, such as sabinene and *β*-phellandrene, and oxygen-containing monoterpenes represented by 4-terpineol. The differences and relationships of these EOs cannot be easily seen from these complex data. Thus, a hierachical cluster analysis was conducted using SPSS (version 17.0) to investigate the similarities and differences among these extracts in EO profiles. The variables were the chemical components and contents of EOs of *C. lansium* seeds. The clustering results are shown in Fig. 2.

Samples were clustered into two clusters: QZ1, QZ2 and other samples. Geographically, QZ1 and QZ2 were both located at 110.02°E, 18.90°N, which were closest to the tropics. This result indicates that the difference between the tropics and subtropics is the greatest. Based on the cluster analysis, three clusters were classified. Table 3 shows the variations in some important volatile components (%) of EOs of *C. lansium* seeds in the three clusters.

**Table 3:** Variations in some important volatile components (%) of EOs of *C. lansium* seeds in the three clusters.

Components	Cluster I						Cluster II					Cluster III	
	HK3	HK4	HK1	HK2	QH1	TC2	LG1	TC1	WC1	QZ3	LG2	QZ1	QZ2
Pinene	1.98	1.48	1.49	1.51	1.53	3.20	**2.62**	**2.30**	**2.27**	**1.22**	**2.00**	2.04	5.10
Sabinene	47.37	49.85	50.01	51.09	52.16	54.28	**39.42**	**37.92**	**42.27**	**42.10**	**43.20**	27.70	33.24
*β*-phellandrene	16.12	14.97	16.19	14.60	15.36	13.39	**21.35**	**23.94**	**23.81**	**25.44**	**25.16**	33.24	25.91
*γ*-terpinene	2.85	2.31	3.37	1.81	2.47	2.30	**2.82**	**2.30**	**2.72**	**2.69**	**0.89**	1.79	2.00
4-terpineol	9.92	9.37	11.28	11.83	7.94	7.27	**8.24**	**7.07**	**7.35**	**8.82**	**6.33**	6.66	6.98
*α*-bergamotene	2.07	2.35	0.29	0.63	2.71	1.45	**0.81**	**2.01**	**2.20**	**4.10**	**1.78**	2.94	0.46

The dominant EOs in each of the three clusters are listed below:

Cluster I: sabinene. HK3, HK4, HK1, HK2, QH1 and TC2 belonged to this cluster. Sabinene in this cluster had a high percentage of almost 50%. The percentages of *β*-phellandrene ranged from 13.39% to 16.19%. HK3, HK4, HK1 and HK2 were classified into a small cluster, which contained more 4-terpineol with relative percentages of 9.37%, 9.92%, 11.28% and 11.83%, respectively. This result is probably because the four places are geographically close (both are in Haikou city). QH1 and TC2, which are both Hainan local wampees, were classified into a small cluster.

Cluster II: sabinene and *β*-phellandrene. These samples were LG1, TC1, WC1, QZ3 and LG2. Although sabinene was the most predominant component in this cluster, with a relative percentage of approximately 40%, the *β*-phellandrene percentage was relatively higher than that in cluster I (more than 20%). LG1 and TC1, chicken heart wampees were classified into a small cluster. WC1, QZ3 and LG2, Hainan local wampees, were classified into a small cluster. Hence, we infer that although the cultivar was not a very clear cause of the different components and contents, some relationships were evident.

Cluster III: *β*-phellandrene. QZ1 and QZ2 were classified into this cluster. They clustered differently from other samples, possibly because QZ1 and QZ2 are closest to the tropics. The percentages of *β*-phellandrene were the highest in all samples, at 33.24% and 25.91%, respectively. They contained similar percentages of sabinene, with 27.70% in QZ1 and 33.24% in QZ2.

### Antifungal activity

The antifungal activity of EOs of *C. lansium* seeds was examined using the filter paper disc diffusion method. Since sabinene, *β*-phellandrene and 4-terpineol are the main components in these EOs, the antifungal activities of three pure chemicals were also examined. As shown in Table 4, the EOs inhibited the growth of all seven strains tested in our study. The inhibition zone diameters of EOs, sabinene, *β*-phellandrene, 4-terpineol and FLZ were in the ranges of 9.4–23.4 mm, 13.7–25.7 mm, 15.6–22.1 mm, 26.7–35.6 mm, and 0–35.3 mm, respectively. Statistical analysis results showed that there was significant difference between FLZ and EOs of *C. lansium* seeds (*P* < 0.05). In all samples and chemicals, 4-terpineol exhibited the greatest antifungal effect against all strains.

**Table 4:** Antifungal activity of EOs of *C. lansium* seeds and three pure chemicals.

Strains	Zone of inhibition (mm)^a^																
	FLZ^b^	sabinene	*β*-phellandrene	4-terpineol	HK4	HK3	HK1	HK2	QH1	TC2	LG1	TC1	WC1	QZ3	LG2	QZ1	QZ2
*C. albliacns* ATCC 10231	34.2 ± 0.7	13.7 ± 0.6	15.9 ± 0.3	35.6 ± 0.5	12.4 ± 0.3	12.4 ± 0.5	12.5 ± 0.8	12.6 ± 0.7	11.4 ± 0.5	11.3 ± 0.4	10.4 ± 0.5	10.0 ± 0.3	11.4 ± 0.3	12.2 ± 0.2	11.4 ± 0.4	9.4 ± 0.4	9.9 ± 0.4
*C. albicnas* 27	0	14.7 ± 0.4	16.1 ± 0.2	35.5 ± 0.6	13.7 ± 0.6	13.8 ± 0.2	14.7 ± 0.4	14.6 ± 0.7	13.5 ± 0.5	13.1 ± 0.2	11.6 ± 0.3	11.3 ± 0.4	13.2 ± 0.3	14.3 ± 0.3	13.2 ± 0.5	11.4 ± 0.3	11.3 ± 0.1
*C. krusei* ATCC 6258	12.3 ± 0.2	25.7 ± 0.6	17.4 ± 0.4	37.7 ± 0.3	22.2 ± 0.3	21.8 ± 0.3	23.4 ± 0.3	23.0 ± 0.4	22.4 ± 0.4	22.1 ± 0.6	19.4 ± 0.5	18.8 ± 0.7	18.3 ± 0.4	19.5 ± 0.5	18.4 ± 0.2	17.8 ± 0.5	17.7 ± 0.3
*C.glabrata* CMCC(F) c6e	23.0 ± 0.1	22.3 ± 0.2	22.1 ± 0.2	28.1 ± 0.2	20.1 ± 0.3	19.9 ± 0.6	20.0 ± 0.6	19.9 ± 0.6	17.0 ± 0.5	16.8 ± 0.3	17.8 ± 0.4	16.9 ± 0.5	16.9 ± 0.4	18.0 ± 0.3	16.6 ± 0.5	16.7 ± 0.4	19.7 ± 0.7
*C. parapsilosis* ATCC 22019	32.2 ± 0.5	15.0 ± 0.4	15.6 ± 0.6	33.6 ± 0.5	12.2 ± 0.5	12.1 ± 0.5	12.2 ± 0.3	11.9 ± 0.4	12.2 ± 0.2	12.1 ± 0.2	11.2 ± 0.2	11.4 ± 0.3	11.2 ± 0.2	11.9 ± 0.3	11.6 ± 0.3	11.1 ± 0.2	11.1 ± 0.3
*C. tropical* CMCC(F) c2f.	35.3 ± 0.4	14.9 ± 0.3	15.6 ± 0.5	27.4 ± 0.4	13.6 ± 0.5	12.6 ± 0.6	13.2 ± 0.3	12.6 ± 0.5	12.1 ± 0.1	12.2 ± 0.2	12.8 ± 0.5	13.0 ± 0.5	12.9 ± 0.5	12.5 ± 0.5	12.5 ± 0.3	12.6 ± 0.7	12.4 ± 0.3
*C. tropical* 13	0	14.9 ± 0.4	15.4 ± 0.5	26.7 ± 0.4	11.4 ± 0.2	10.9 ± 0.2	10.8 ± 0.3	10.8 ± 0.4	10.4 ± 0.4	9.5 ± 0.5	9.5 ± 0.4	11.3 ± 0.2	10.4 ± 0.1	10.5 ± 0.3	10.7 ± 0.4	10.6 ± 0.4	11.2 ± 0.3

^a^Values represent the means of three independent replicates ± SD; concentrations: EOs:10 μL/disc; ^b^FLZ: Fluconazole, 25 μg/disc.

Table 4 shows that there were differences in the antifungal activity efficacy among different types of strains. The antifungal activity of EOs against *C. glabrata* and *C. krusei* was higher than that of the other five *Candida* strains and there were signifcant differences (*P* < 0.05).

For *C. albicans,* the antifungal activity efficacies of both EOs and the constituent chemicals against the FLZ-resistant strain (*C. albicans* 27) were better than those against the FLZ-sensitive strain (*C. albicans* ATCC 10231). The efficacy order of the three pure chemicals was as follows: 4-terpineol > *β*-phellandrene > sabinene. Among the EOs, HK1, HK2, HK3 and HK4 in cluster I exhibited the greatest antifungal effects and were rich in 4-terpineol. The antifungal activity of QZ3 in cluster II was also higher, which may be because it contained higher contents of 4-terpineol and *β*-phellandrene. Cluster III showed the weakest effect against *C. albicans.*

The EOs *C. lansium* seeds exhibited the greatest antifungal effect against *C. krusei*, with inhibition zone diameters of 17.7–23.4 mm. The efficacy order was 4-terpineol > sabinene > *β*-phellandrene > cluster I > cluster II > cluster III > FLZ. In EOs, cluster I exhibited the greatest antifungal effect, which may be due to the high contents of 4-terpineol and sabinene. The percentage of 4-terpineol and sabinene in cluster III were relatively lower. Therefore, even if the percentage of *β*-phellandrene was the highest, the antifungal effect of cluster III was still the worst against *C. krusei*.

For *C. glabrata,* the inhibition zone diameters of FLZ, sabinene, *β*-phellandrene, and 4-terpineol were 23.0 mm, 22.3 mm, 22.1 mm, and 28.1 mm, respectively. Statistical analysis results showed that the antifungal activity efficacies of sabinene and *β*-phellandrene were similar (*P* = 0.687). The antifungal activity of 4-terpineol was higher than FLZ and there was significant difference (*P* = 4.99 × 10^−17^). EOs in HK1-4 and QZ2 showed greater antifungal effects, while QH1, TC1, TC2, WC1 and LG2 showed weaker effects against *C. glabrata*. Considering their compositions, we infer that these differences may be related to the contents of monoterpenes containing oxygen: the higher the contents of monoterpenes containing oxygen, the higher the antifungal effect.

The antifungal activity efficacy order was 4-terpineol > *β*-phellandrene > sabinene against *C. parapsilosis,* with inhibition zone diameters of 33.6 mm, 15.6 mm, and 15.0 mm, respectively. Among the EOs of *C. lansium* seeds, the antifungal activities of clusters I and II were similar, and cluster III showed the weakest effect against *C. parapsilosis.*

For *C. tropicalis,* the antifungal activity of 4-terpineol was also greatest in these samples and chemicals. The inhibition zone diameters of EOs were in the range of 12.1–13.6 mm against the sensitive strain (*C. tropicalis* CMCC(F) c2f.) and 9.5–11.4 mm against the resistant strains (*C. tropicalis* 13). There was no significant difference in the antifungal effects among the three clusters (*P* > 0.05).

## Discussion

In a previous study, EOs of *C. lansium* from Changjiang County, Hainan, China were obtained by hydrodistillation and were analysed by GC-MS^24^. The oil yield was 0.5% (*v/w*). Twenty-six components were identified, and the main components identified in the seed EO were phellandrene (54.8%), limonene (23.6%) and *p*-menth-1-en-4-ol (7.5%). Using the same hydrodistillation method, fifty-three components were identified in both fresh and dried fruit samples from Thailand^25^. The main components were sabinene (33.68–66.73%), *α*-pinene (9.57–13.35%) and 1-phellandrene (5.77–10.76%). Comparison revealed that the components and percentages were different from different areas. The reasons for the differences may be due to the fruit containing not only seeds but also pulp and pericarp.

The oil yield was 0.1% (*v/w*) in the supercritical fluid extraction (SFE) of *C. lansium* fruits from Guangdong, China^26^. Thirty-six components were identified, and the main components were 4-terpineol (26.94%), *γ*-terpinene (14.39%), *β*-phellandrene (8.24%) and sabinene (5.58%). Chokeprasert et al.^27^ analysed the volatile components of *C. lansium* fresh seeds in Thailand by headspace (HS) GC–MS. Twenty-five components were identified in the seeds, and the major components were sabinene (83.56%) and *α*-pinene (4.26%). In our previous study, volatile components of *C. lansium* seeds from different areas in Hainan, China, were detected using ionic liquid-based HS-GC-MS^21^. Thirty-three components were detected. The volatile components were rich in sabinene (17.76–43.81%) and were accompanied by *β*-bisabolene (5.83–22.25%), caryophyllene (6.75–16.62%) and *β*-sesquiphellandrene (3.20–22.25%). The components and percentages obtained by SFE and HS were quite different from those obtained by hydrodistillation.

In the present study, the differences in the compositions of EOs of *C. lansium* seeds from different areas and cultivars were analysed and compared for the first time. Cluster analysis results showed that the differences in the components were most significantly contributed by different harvest areas, followed by different cultivar. The main components were sabinene, *β*-phellandrene, and 4-terpineol.

Few studies have reported the antifungal activity of *C. lansium* extracts against *Candida* strains. Xu et al.^28^ reported that extracts of pericarps of *C. lansium* using 95% alcohols had a significant inhibitory effect on *C. albicans*, with an inhibition zone diameter of 16.8 mm by the filter paper disc diffusion method. However, the components of these extracts have not yet been elucidated. In our previous study^29^, we determined the chemical compositions of *C. lansium* EOs of pericarps and leaves by GC–MS. Their potential antifungal effects against five *Candida* species (*C. albicans*, *C. tropicalis*, *C. glabrata*, *C. krusei* and *C. parapsilosis*) were evaluated using the disc diffusion method. *C. lansium* EOs of pericarps displayed higher antifungal activities than EOs of leaves. Higher contents of *β*-phellandrene, *β*-sesquiphellandrene and *β*-bisabolene in EOs of pericarps were likely responsible for the high antifungal activity.

In previous reports, Abdelli et al.^30^ studied the chemical compositions and antibacterial activities of the leaves and berries of Algerian *Juniperus phoenicea* EOs and found that higher contents of *β*-phellandrene and *α*-pinene were positively correlated with the antibacterial activity. A study of the antibacterial activity of *Piper* species EOs from distinct rainforest areas in southeastern Brazil showed that was *β*-phellandrene was responsible for the higher antibacterial effects^31^. *Melaleuca alternifolia* (tea tree) EO was particularly rich in 4-terpineol (40.44%) and *γ*-terpinene (19.54%) and showed significant antifungal activity against all *Candida* strains tested^32^. The antifungal activity could, in part, be attributed to the major component 4-terpineol.

In this study, the EOs of *C. lansium* seeds showed significant activity against all the strains. There were differences among the different types of *Candida* strains. Notably, they were active towards FLZ-resistant strains (*C. albicans* 27, *C. tropicalis* 13). Moreover, the antifungal activity of EOs against *C. glabrata* and *C. krusei* was higher than that of the other five *Candida* strains, and *C. glabrata* and *C. krusei* were the main *Candida* spp. that might have fluconazole resistance (with lower inhibition zone diameters). Therefore, it comes to a conclusion that the natural EOs of *C. lansium* seeds might be more effective against FLZ-resistant strains. Comprehensive analysis illustrated the importance of sabinene, *β*-phellandrene and 4-terpineol to antifungal activity, and there might be synergism between the pure chemicals. In addition, antifungal activity may not entirely depend on the main chemical components. Other chemical components present at lower concentrations may also have antifungal activity or may have synergistic effects with other components. More detailed in vivo studies are still necessary.

In summary, this study identified a total of forty-six components in the EOs of *C. lansium* (Lour.) Skeels seeds by GC–MS. The main components were sabinene, *β*-phellandrene, and 4-terpineol. Cluster analysis results showed that the differences in the components were most significantly contributed by different harvest area, followed by different cultivar. EOs of *C. lansium* seeds showed significant antifungal activity against all seven tested *Candida* strains. Through comprehensive comparison and analysis of chemical composition and antifungal activity, higher contents of sabinene, *β*-phellandrene and 4-terpineol may be positively correlated with antifungal activity. There were differences in the antifungal activity efficacies among the different types of strains. Notably, EOs of *C. lansium* seeds were active towards FLZ-resistant strains. This study may provide a basis for the development of EOs *C. lansium* seeds as new antifungal agents with high efficiency, broad-spectrum activity, low toxicity and low cost. It may also provide new solutions for clinical infections caused by drug resistance problems.

## Materials and methods

### Plant materials and chemicals

The fruits of *C. lansium* were collected from Hainan, China, in May and June 2020. The details of the source, location, and cultivar of *C. lansium* samples employed in this study are listed in Table 5. Experimental research and field studies on plants were carried out in accordance with relevant institutional, national, and international guidelines and legislation. The permissions for collection of seed specimens were approved by the planters. All samples were identified by Professor Jianping Tian (Hainan Medical University) and were deposited in the Public Research Laboratory of Hainan Medical University (Hainan, China). C8-C40 *n*-alkanes (500 mg·L^−1^ in hexane) were purchased from America o2si (South Carolina, USA), sabinene (75%) from Sigma-Aldrich (Shanghai) Trading Co., Ltd. (Shanghai, China), *β*-phellandrene (96%) from Toronto Research Chemicalstrc (Toronto, Canada) and 4-terpineol (98%) from Shanghai Aladdin Biochemical Technology Co., Ltd. (Shanghai, China). Other chemicals and their suppliers are listed as follows: Sabouraud’s dextrose agar (SDA) and Mueller–Hinton agar (MHA) powder (Guangdong Huankai Microbial Sci. & Tech. Co., Ltd., Guangdong, China), fluconazole (FLZ) (Aladdin, Shanghai, China), and C8-C40 *n*-hexanes (chromatographic purity, Aladdin).

**Table 5:** Source, location, and cultivar of *C. lansium* samples.

No	Source	Location (longitude, latitude)	Cultivar ^a^
HK1	Longqiao Town, Haikou City	110.36, 19.91	HLW
HK2	Meixiao Village, Haikou City	110.34, 19.85	CHW
HK3	Longquan Town, Haikou City	110.36, 19.85	HLW
HK4	Ruxu Village, Haikou City	110.26, 19.89	CHW
LG1	Heshe Village, Lingao City	109.72, 19.60	CHW
LG2	Heshe Village, Lingao City	109.72, 19.60	HLW
WC1	Dawei Village, Wenchang City	110.75, 19.87	HLW
QH1	Hongzhuang Brigade, Qionghai City	110.51, 19.22	HLW
QZ1	Heping Town, Qiongzhong City	110.02, 18.90	HLW
QZ2	Heping Town, Qiongzhong City	110.02, 18.90	CHW
QZ3	Yangjiang Form, Qiongzhong City	109.84, 19.24	HLW
TC1	Zhongkun Form, Tunchang City	109.95, 19.32	CHW
TC2	Zhongkun Form, Tunchang City	109.95, 19.32	HLW

^a^*C. lansium*with globose fruits is named Hainan local wampee (HLW): the berry fruit is approximately 1.5–2.5 cm in diameter. *C. lansium* with broadly ovoid fruits is named chicken heart wampee (CHW), with berries approximately 1.5–2.5 × 2.5–3.5 cm in diameter.

### Strains and culture media

The *Candida* strains included the standard reference strains (*C. albicans* ATCC 10231, *C. parapsilosis* ATCC 22019, *C. krusei* ATCC 6258, *C. glabrata* CMCC(F) c6e and *C. tropicalis* CMCC(F) c2f.) and clinical strains (*C. albicans* 27, *C. tropical* 13). *C. albicans* ATCC 10231 and *C. parapsilosis* ATCC 22019 were obtained from Guangdong Huankai Microbial Sci. & Tech. Co., Ltd. (Guangdong, China), *C. krusei* ATCC 6258 from Wenzhou Kangtai Biotechnology Sci. & Tech. Co., Ltd. (Wenzhou, China), *C. glabrata* CMCC(F) c6e and *C. tropicalis* CMCC(F) c2f. from National Center for Medical Culture Collection (Beijing, China). The clinical strains were obtained from the oral mucosa of patients with oral candidiasis at Hainan Wenchang General Hospital and Sanya General Hospital, located in Wenchang and Sanya, respectively, China. The sampling methods were carried out in accordance with relevant guidelines and regulations. All experimental protocols were approved by Hainan Medical University and informed consent was obtained from all subjects. The clinical isolates were previously identified based on ITS gene sequence analysis. The obtained yeast isolates were stored in a − 80 °C freezer until use. The culture media used were SDA and MHA.

### Instrumentation

Measurements were carried out on a GC–MS-QP2010-Plus system (Shimadzu, Kyoto, Japan) in electron impact ionization (EI) mode. The column was DB-5MS fused silica (30 m × 2.5 mm; 0.25 μm film thickness) from Agilent, USA. Other instrumentation included an FW-80 high-speed grinder (Tianjin Taisite Instrument Co., Ltd, Tianjin, China), an AL104 electronic balance (METTLER TOLEDO Instrument Co., Ltd., Shanghai, China), a Tanon-4100 automatic digital gel image analysis system (Shanghai Tianlong Technology Co., Ltd., Shanghai, China), and an SPX-250B-Z biochemical incubator (Shanghai Bo Xun Industrial Co., Ltd., Shanghai, China)^29^.

### EO extraction

The seeds of *C. lansium* were extracted from the fruits, washed and then dried in an oven at 40–50 °C. Then, the seeds were roughly crushed with a grinder and soaked in water with the ratio of material to solvent 1:8 (*w/v*) for 5.0 h prior to hydrodistillation. The EOs were extracted by hydrodistillation for 3.0 h in a Clevenger-type apparatus. The volume of extracted EOs was read from the scale of apparatus. The yield was calculated by the ratio of extracted volume to weight. The obtained EOs were stored at 4 °C in an airtight container and dried using anhydrous sodium sulfate before being analysed.

### GC–MS analysis

Samples were analysed by the GC–MS EI method with a DB-5MS column. Helium was used as a carrier gas at a flow rate of 1.0 mL·min^−1^ in split mode (1:20). The column temperature was maintained at 60 °C for 3 min and then programmed to 140 °C at a heating rate of 5 °C·min^−1^, where it was held for 2 min, after which it was increased to 210 °C at a rate of 10 °C·min^−1^ for the remaining 7 min. The temperatures of both the injector and the connector were maintained at 250 °C. The operating parameters of MS were EI mode at 70 eV with a mass scanning range of 50–500 amu and a source temperature of 280 °C. C8-C40 *n*-alkanes were used as reference points in the calculation of relative retention indexes (RIs). The percentages of compositions were obtained from the electronic integration of peak areas. The identity of each compound was determined by comparing its RI to *n*-alkanes and the NIST library database (NIST08 and NIST08s). The percentage of composition was computed from peak areas without applying correction factors^29^.

### Paper disc diffusion method

The antifungal activity of the EOs of *C. lansium* seeds was first investigated using the paper disc diffusion method^33^. All strains were streaked onto the SDA plates. A single colony was streaked again to ensure the viability and purity of the strains. Then about 5 single colonies were picked up into 5 mL saline solution. Microbial suspensions of 0.5 McFarland standard were prepared equivalent to approximately 10^6^ CFU·mL^−1^ yeast and evenly dispensed onto the MHA plates to form the microbe lawn. Pieces of papers were placed on the medium with sterile tweezers and keep the space between the paper more than 24 mm. The diameters of MHA plates and the paper were 120 mm and 6 mm, respectively. The antifungal drug FLZ (2.5 mg·mL^−1^) was used as a positive control. Ten microlitres of EOs of *C. lansium* seeds, the constituent chemicals (sabinene, *β*-phellandrene and 4-terpineol) and positive control were added onto the paper. The plates were incubated at 30 °C for 24 to 48 h. The antifungal activity of the samples was evaluated by measuring zones of inhibition of microbial growth surrounding the paper discs. The zones of inhibition corresponding to diameters were measured with an antibiotic zone scale (Cecon-Brasil) in mm. All the experiments were carried out in triplicate.

### Statistical analysis

Data analysis was performed using SPSS software (version 17.0). Data represent the mean ± standard deviation (SD) from at least 3 distinct experiments. Statistical differences were determined using a 2-sample equal variance Student’s *t* test. Statistical significance was defined as *P < 0.05. GC–MS data and chromatograms were processed with Shimadzu GC–MS Solution software.
